# Regulation of C3 Activation by the Alternative Complement Pathway in the Mouse Retina

**DOI:** 10.1371/journal.pone.0161898

**Published:** 2016-08-26

**Authors:** Jennifer A. E. Williams, Dimitris Stampoulis, Chloe E. Gunter, John Greenwood, Peter Adamson, Stephen E. Moss

**Affiliations:** 1 Department of Cell Biology, UCL Institute of Ophthalmology, 11–43 Bath Street, London EC1V 9EL, United Kingdom; 2 Ophthiris Discovery Performance Unit and Department of Laboratory Animal Science, GlaxoSmithKline, Medicines Research Centre, Gunnelswood Road, Stevenage, Herts SG1 2NY, United Kingdom; University of Cologne, GERMANY

## Abstract

The purpose of this study was to examine the retinas of mice carrying hemizygous and null double deletions of *Cfb*^-/-^ and *Cfh*^-/-^, and to compare these with the single knockouts of *Cfb*, *Cfh* and *Cfd*. Retinas were isolated from wild type (WT), *Cfb*^-/-^*/Cfh*^-/-^, *Cfb*^-/-^*/Cfh*^+/-^, *Cfh*^-/-^*/Cfb*^+/-^, *Cfb*^-/-^, *Cfh*^-/-^ *Cfd*^-/-^, and *Cfd*^+/-^ mice. Complement proteins were evaluated by western blotting, ELISA and immunocytochemistry, and retinal morphology was assessed using toluidine blue stained semi-thin sections. WT mice showed staining for C3 and its breakdown products in the retinal vasculature and the basal surface of the retinal pigment epithelium (RPE). *Cfb*^-/-^ mice exhibited a similar C3 staining pattern to WT in the retinal vessels but a decrease in C3 and its breakdown products at the basal surface of the RPE. Deletion of both Cfb and Cfh restored C3 to levels similar to those observed in WT mice, however this reversal of phenotype was not observed in *Cfh*^-/-^/*Cfb*^+/-^ or *Cfb*^-/-^/*Cfh*^+/-^ mice. Loss of CFD caused an increase in C3 and a decrease in C3 breakdown products along the basal surface of the RPE. Overall the retinal morphology and retinal vasculature did not appear different across the various genotypes. We observed that C3 accumulates at the basal RPE in *Cfb*^-/-^, *Cfb*^-/-^*/Cfh*^-/-^, *Cfb*^-/-^/*Cfh*^+/-^, *Cfd*^-/-^ and WT mice, but is absent in *Cfh*^-/-^ and *Cfh*^-/-^/*Cfb*^+/-^ mice, consistent with its consumption in the serum of mice lacking CFH when CFB is present. C3 breakdown products along the surface of the RPE were either decreased or absent when CFB, CFH or CFD was deleted or partially deleted.

## Introduction

Dysregulation of the complement system is a recognised characteristic of patients with age-related macular degeneration (AMD). However, AMD is a multifactorial disease and isolating the specific contributions of individual complement proteins to disease pathology is not straightforward. One approach to studying the role of the complement system in the maintenance of retinal health is to evaluate and compare the retinal phenotype of mice carrying mutations or deletions of specific complement genes.

In AMD there are several complement gene single nucleotide polymorphisms (SNP) known to associate with increased susceptibility to or protection against AMD. The complement factor H (CFH) gene contains the most common complement-associated high risk SNP for AMD that changes a tyrosine at position 402 to a histidine [1–4]. CFH is a fluid-phase regulator of the alternative pathway of complement activation, exerting its activity in three ways, by i) preventing C3 convertase formation, ii) promoting its dissociation and iii) participating in the breakdown of the active split product C3b. Formation of the C3 convertase is driven by complement factor B (CFB), and consequent downstream activation of C5 and generation of the membrane attack complex. In previous studies we showed that *Cfh*^-/-^ mice exhibit visual dysfunction at 2 years, with C3 and autofluorescent debris accumulating at Bruch’s membrane, disorganisation of retinal pigment epithelial (RPE) cell organelles and thinning of Bruch’s membrane [5]. Early compensatory signs of complement dysregulation are evident at 1 year in *Cfh*^-/-^ mice where CD59 mRNA expression in the neuroretina is reduced [6] and CD55 (decay-accelerating factor) expression in Müller cells is up-regulated [7].

Recently it has been shown that the Y402H SNP in CFH affects its binding to the lipid peroxidation product malondialdehyde, which accumulates with age [8]. SNPs that provide protection against AMD have also been identified such as the R32Q variant of complement factor B (CFB) [9]. CFB is expressed by the RPE [10] and upon down-regulation by siRNA or complete gene knock-out in mice, has been shown to provide protection against choroidal neovascularisation after laser burn of Bruch’s membrane [11,12]. Complement factor D (CFD), which is a serine protease required for the generation of the C3 convertase, is currently a focus of interest due to on-going clinical trials of the function-blocking antibody Lampalizumab in dry AMD [13,14], and *Cfd* gene knockout has been shown to be protective in a mouse model of light-induced phototoxicity [15]. However phenotypic characterisation of the *Cfb*^-/-^ and *Cfd*^-/-^ retinas has not been reported.

A caveat of studies using *Cfh*^-/-^ mice is that their serum is almost completely depleted of C3 since without CFH there is no fluid phase regulator to prevent its breakdown. Furthermore, these mice also lack CFB as this is consumed in the breakdown of C3. Therefore these mice are close to being a CFH, C3, CFB triple knock-out. Unlike the *Cfh*^-/-^ mice, *Cfb*^-/-^*/Cfh*^-/-^ double knockout mice would be expected to have normal to higher levels of C3 since without CFB, C3 cannot be broken down via the alternative pathway. We would also expect this to be the case in *Cfd*^-/-^ mice. Without CFB or CFD we would not expect to see C3 breakdown products since the C3 convertase cannot form without both activators. In this study, we address these questions by characterising the retinas of *Cfb*^-/-^, *Cfh*^-/-^ and *Cfd*^-/-^ mice at 12 months to identify signs of retinal abnormalities, and for the first time report the phenotype of the *Cfb*^-/-^*/Cfh*^-/-^ double knock-out mouse retina, with *Cfh*^-/-^/*Cfb*^+/-^ and *Cfb*^-/-^/*Cfh*^+/-^mice examined as a gene dosage control for CFB and CFH.

## Materials and Methods

### Animals

Wild-type, *Cfb*^-/-^, *Cfh*^-/-^, *Cfb*^-/-^*/Cfh*^-/-^, *Cfb*^-/-^*/Cfh*^+/-^ and *Cfh*^-/-^*/Cfb*^+/-^ mice were housed for 12 months at Charles River Laboratories France, Domaine des Oncins, BP 10969592, L’ARBRESLE CEDEX. *Cfd*^+/+^, *Cfd*^+/-^ and *Cfd*^-/-^ mice were housed at GSK. All mice were on the C57/Bl6J background. This study was ethically reviewed and approved by the Institute of Ophthalmology Animal Welfare and Ethical Review Board, and carried out in accordance with Animals (Scientific Procedures) Act 1986 and the GSK Policy on the Care, Welfare and Treatment of Animals. All mice were sent to UCL where they were culled by a rising concentration of CO_2_ with subsequent cervical dislocation and the eyes removed immediately.

### Semithin sections

Eyes were fixed in Karnovsky’s fixative (3% glutaraldehyde (EM grade-TAAB, G002), 1% paraformaldehyde in 0.07 M sodium cacodylate (Agar Scientific, R1104), pH 7.4) for 2 h at RT. Sectioning and staining were performed as previously described [16].

### Immunofluorescence

After cervical dislocation, eyes were enucleated and immediately placed in 2% paraformaldehyde (1x PBS) for 7 min on ice. Eyes were then transferred to 2 x PBS for up to 30 min on ice prior to dissection. Eyes were then fixed again in 4% paraformaldehyde for 30 min at RT and subsequently cryopreserved in OCT embedding matrix and cut into 12 μm sections. Sections were blocked and permeabilised in PBS containing 1% bovine serum albumin, 0.5% Triton X-100 and 0.12% sodium azide. Sections were either stained with a C3 antibody conjugated to FITC fluorophore (Cappel, 1:100 dilution) or a C3b/iC3b/C3c antibody (Rat monoclonal antibody, Hycult, 1:50 dilution) overnight at 4°C. Sections stained with C3b/iC3b/C3c antibody were then washed and stained with anti-rat IgG conjugated to AlexaFluor 594 (1:200 dilution) for 1 h at RT. Sections were again washed and incubated with 1μg/ml DAPI for 2 min at RT before mounting in Mowiol mounting medium.

### Retinal wholemounts

Mouse eyes were fixed in 4% paraformaldehyde for 30 min and remained in PBS overnight before dissecting. The neuroretina was peeled away from the RPE and incisions made to flatten before fixing in ice-cold methanol. Flatmounts were blocked and permeabilised for 1h at RT in 2X PBS containing 1% bovine serum albumin, 3% Triton X-100, 0.5% Tween-20 and 0.12% sodium azide. Flatmounts were stained using Collagen IV (Rabbit polyclonal, AbD Serotec, 1:500) antibody overnight at RT, followed by anti-rabbit IgG conjugated to AlexaFluor 488 and anti-rat IgG conjugated to AlexaFluor 594 (1:200 dilution). Sections were again washed and incubated with 1μg/ml DAPI for 2 min at RT before mounting in Mowiol mounting medium. The entire ribbon containing the optic nerve was analysed for each sample. We did not detect noticeable differences in C3 or C3 breakdown products along the ribbon, and therefore took images halfway along on either side of the optic nerve.

### Western blot analysis of mouse C3, CFB, CFH, CFD and C5

Mouse blood was collected by cardiac puncture in the presence of ethylenediamine tetraacetic acid (EDTA) from 6 month old mice of the genotypes listed earlier. Samples were chilled on ice and then plasma-separated by centrifugation at 4°C for 20 min. Proteins were separated using sodium dodecyl sulphate-polyacrylamide gel electrophoresis (SDS-PAGE): 4–12% gel under reducing conditions for C3, CFB, CFH and CFD, and 4–12% gel under non-reducing conditions for C5. PVDF membranes were blocked in 7% w/v non-fat dry milk in PBS. The buffer used for diluting the primary and secondary antibodies was 3.5% w/v non-fat dry milk in 0.025% Tween20/PBS. All the intermediate washing steps were performed in 0.05% Tween20/PBS. Antibodies used were peroxidase-conjugated goat anti-mouse complement C3 (product no. 55557; MP Biomedicals, 1:500), goat antisera to human CFH (product no. A312; Quidel, 1:500), goat antisera to human CFB (product no. A311; Quidel, 1:500), polyclonal sheep immunoglobulin (IgG) to mouse CFD (product no. AF5430; R&D Systems, 1:500) and goat antisera to human C5 (product no. A306; Quidel, 1:500). Secondary antibodies were polyclonal rabbit anti-goat HRP (product no. P0449; Dako, 1:1000), polyclonal rabbit anti-sheep HRP (product no. P0163; Dako, 1:1000), and peroxidase AffiniPure sheep anti-mouse IgG (product no. 515-035-071, Jackson Immunoresearch, 1:1000). Blots were visualized using Amersham enhanced chemiluminescence (ECL) Western Blotting Detection Reagent (product no. RPN2106, GE Healthcare Life Sciences).

### ELISA for plasma C3

Microtiter plates were coated overnight at 4°C with polyclonal goat IgG to mouse complement C3 (product no. 55463, MP Biomedicals, 1:8000) in PBS. The plates were then washed in 0.2% Tween20/PBS and blocked with 2% BSA in 0.2% Tween20/PBS at RT for 60 min. After washing twice, mouse plasma samples were loaded at 1:6000 and 1:12000 dilutions and left for 60 min at RT. Mouse C3 was detected with peroxidase-conjugated goat IgG fraction to mouse complement C3 (product no. 55557, MP Biomedicals, 1:25000) in 0.2% Tween20/PBS. Plates were developed using TMB substrate Reagent Pack (product no. DY999, R&D Systems). The concentration of plasma C3 was estimated by reference to a calibration curve constructed with 11 serial dilutions (1:2) of pooled mouse plasma of known C3 concentration (220 μg/ml). Plasma samples from 5–6 mice were tested for each genotype. Unpaired, two-tailed Student’s t-tests were applied to data.

## Results

**Fig 1:**
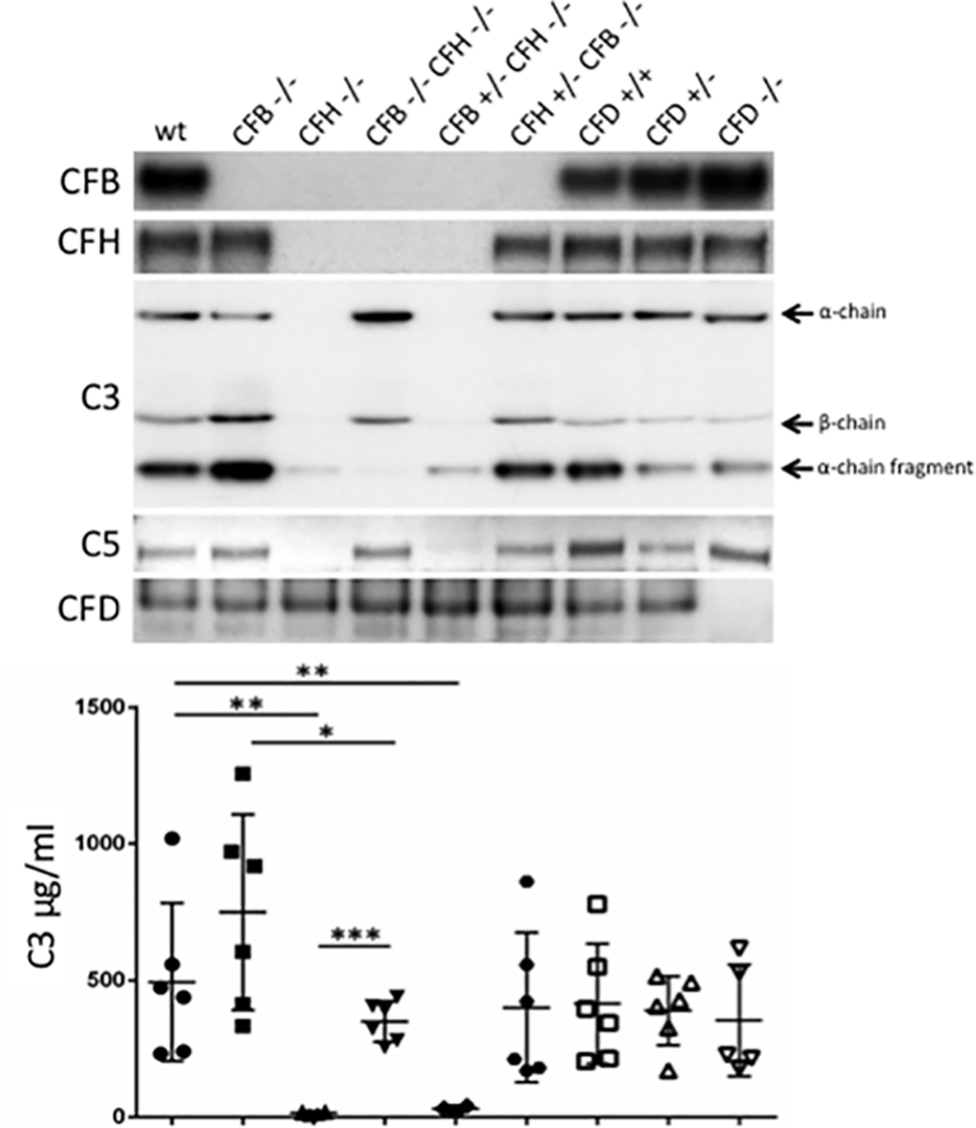
Expression of complement proteins in multiple complement gene knock-out mice. Plasma samples were taken from mice at age 6 months corresponding to the genotypes indicated. For western blotting, 1 μl whole plasma was analysed using specific antisera as detailed in the Materials and Methods. Representative blots are shown for each protein. For C3 ELISA, plasma samples from 5–6 mice were analysed per genotype. Data are means ± S.D, unpaired, two-tailed Student’s t-tests were applied to data. *P = < 0.05, **P = < 0.01, ***P = < 0.001.

The three pathways of complement activation converge upon C3, which is the central component of the complement cascade. In order to determine the impact of null mutations in the *Cfb*, *Cfh*, and *Cfd* genes on the levels of circulating complement proteins, plasma samples from the corresponding mutant mice were examined by western blotting and ELISA (Fig 1). As expected, gene knockout of *Cfb*, *Cfh* and *Cfd* resulted in the absence of the cognate gene product from the plasma. However, whilst CFB was not detected in the plasma of *Cfb*^-/-^ mice, it was also absent in samples from the *Cfh*^-/-^, *Cfb*^-/-^/*Cfh*^-/-^, *Cfh*^-/-^*/Cfb*^+/-^ and *Cfb*^-/-^*/Cfh*^+/-^ strains. In the *Cfd*^+/-^ and *Cfd*^-/-^ mice we observed that CFB levels were elevated relative to the WT *Cfd*^+/+^ mice. CFH was not present in detectable levels in the plasma of the *Cfh*^-/-^ mice and neither was CFH detected in the plasma of any of the other strains carrying a homozygous *Cfh* deletion. Restoring CFH expression to heterozygous levels in the *Cfb*^-/-^*/Cfh*^+/-^ mice resulted in detectable but slightly reduced levels of CFH compared to the WT.

In the *Cfd*^-/-^ and *Cfd*^+/-^ mice, CFH was present with no apparent difference in expression compared to the WT *Cfd*^+/+^. Indeed, CFD was present at similar levels in all tested plasma samples apart from the *Cfd*^-/-^ mice. The presence of plasma C5 was also analysed and the expression pattern across the nine genotypes mirrored that of C3. Thus, C5 was not detected in the *Cfh*^-/-^ mice, but deletion of *Cfb* in order to generate the Cfb^-/-^/Cfh^-/-^ mice restored the expression of C5 to levels comparable to those observed in WT mice. In contrast, in Cfh^-/-^/Cfb^+/-^ mice, decreasing CFB expression to heterozygous levels did not lead to recovery of C5. In the *Cfd*^-/-^ and *Cfd*^+/-^ mice C5 was present but slightly reduced when compared to the WT Cfd^+/+^ mice.

Complement C3 was evaluated by ELISA to obtain actual plasma concentrations, and also by western blotting to visualize the α and ß chains. In the *Cfh*^-/-^ mice C3 was reduced to almost undetectable levels whereas in the *Cfb*^-/-^ mice, levels of C3 were slightly though not significantly elevated compared to WT, consistent with C3 degradation being dependent on CFB. Deletion of *Cfb* in the *Cfh*^-/-^ mice, to generate the *Cfb*^-/-^*/Cfh*^-/-^ mice, reversed the level of C3 in the plasma to that observed in WT mice. However, in *Cfh*^-/-^/*Cfb*^+/-^ mice, decreasing CFB expression to heterozygous levels was insufficient to rescue C3, which remained at almost undetectable levels. In contrast, restoring CFH expression to hemizygous levels in the *Cfb*^-/-^/*Cfh*^+/-^ mice had no effect on plasma C3. Loss or partial loss of CFD in the *Cfd*^-/-^ and *Cfd*^+/-^ mice respectively, caused no significant changes in plasma C3 levels. The effects of these multiple complement gene knockouts on components of the alternative pathway are broadly in line with previous reports [17].

**Fig 2:**
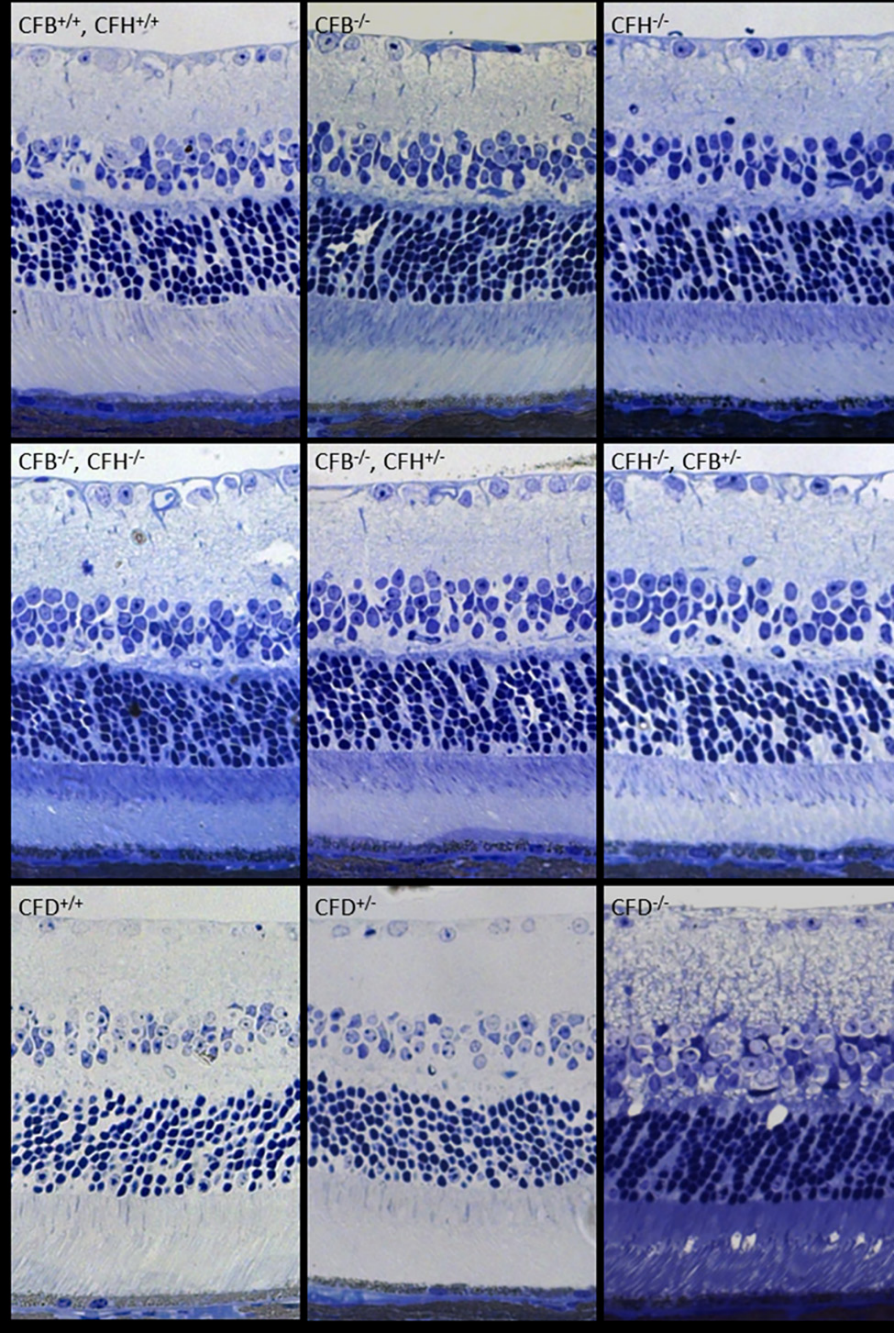
Loss of *Cfb*, *Cfh* or *Cfd* does not affect retinal morphology in one year mice. The images show toluidine blue stained 2 μm semithin sections of mouse retinas of indicated genotypes at 12 months. Scale bar = 50 μm. Images are representative of n = 3.

**Fig 3:**
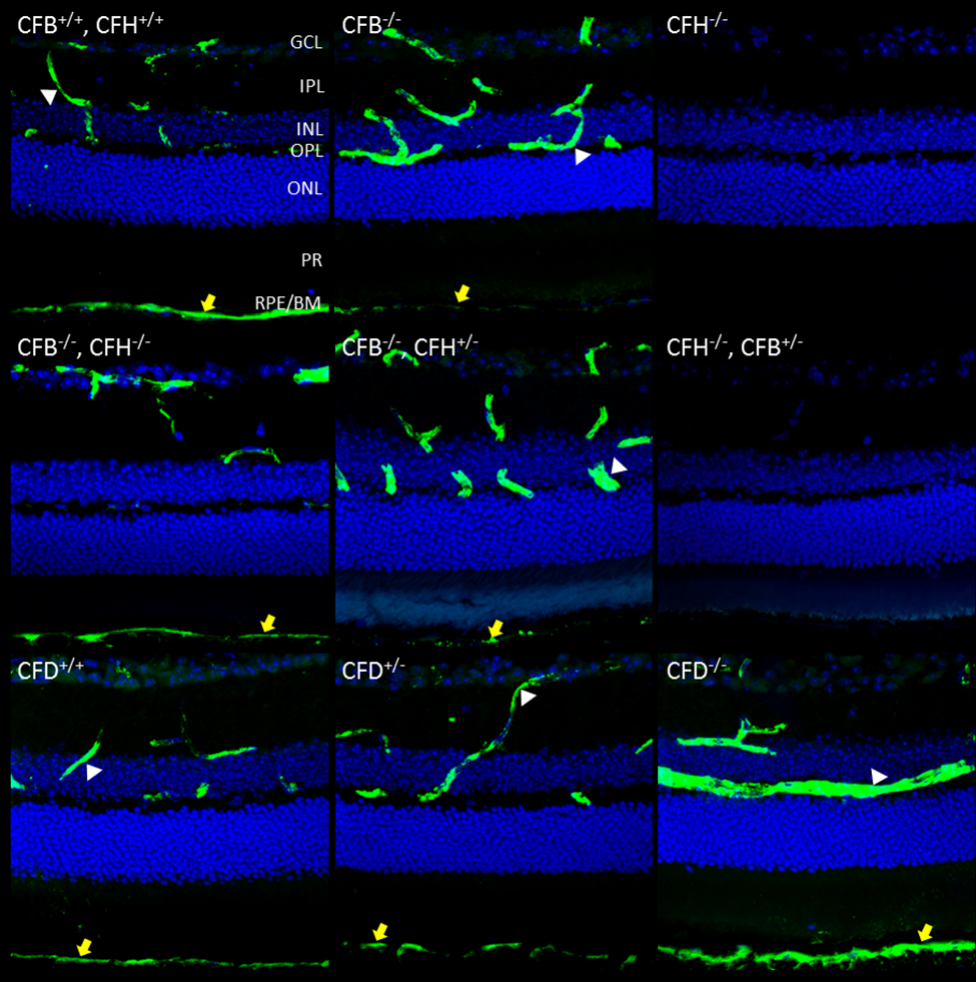
Immunostaining of C3 is restored to wild type levels in the retinal vessels and RPE/Bruch’s space in *Cfh*^-/-^ mice when in the genetic background of *Cfb*^-/-^ but not *Cfb*^+/-^. 12 μm PFA fixed sections were taken of mouse retinas of the indicated genotypes at age 12 months. Sections were stained for C3-FITC (green) and nuclei (blue). Yellow arrows highlight staining in RPE/Bruch’s. Z-stacks were imaged using confocal microscopy and merged to form a maximum intensity projection. Scale bar = 50 μm. Images are representative of n = 6.

In order to identify any gross anatomical differences in retinal morphology caused by the loss of the complement genes under investigation here, semi-thin sections were cut and stained with toluidine blue (Fig 2). As pilot studies across the range of genotypes revealed no obvious changes at 6 months, animals were examined at 12 months to increase the chances of detecting slow onset phenotypic changes. However, all genotypes presented a similar morphology, with no marked differences between them with regard to retinal thickness or numbers of photoreceptor nuclei. We next examined retinal sections for C3 deposition, as accumulation of C3 and its breakdown products is a well-established characteristic of retinas from patients with AMD [18,19]. In WT mice, we observed C3 immunostaining within the retinal blood vessels, corresponding to the circulating pool and, consistent with previous reports, also along the basal surface of the RPE (Fig 3) [5,20]. In *Cfb*^-/-^ mice, C3 staining was evident in the retinal blood vessels and along the basal surface of the RPE but to a markedly lesser extent than that observed in WT mice. As one would expect, given the results in Fig 1, the *Cfh*^-/-^ mouse lacked any C3 staining in the retina, whereas in the *Cfb*^-/-^*/Cfh*^-/-^ mouse this phenotype was reversed such that C3 returned to levels comparable to those observed in WT mice. Heterozygosity for CFH in the *Cfb*^-/-^/*Cfh*^+/-^ mouse led to reduced staining of C3 in the RPE/Bruch’s. In *Cfh*^-/-^*/Cfb*^+/-^ mice, decreasing CFB expression to heterozygous levels was insufficient to rescue C3 stability, which remained absent from the retinal vessels and the basal surface of the RPE. In the *Cfd*^-/-^ mouse, but not the *Cfd*^+/-^, the staining of C3 in the RPE/Bruch’s was notably more intense than in control animals, perhaps because the absence of CFD would result in failure to generate the C3 convertase, leading to accumulation of C3.

**Fig 4:**
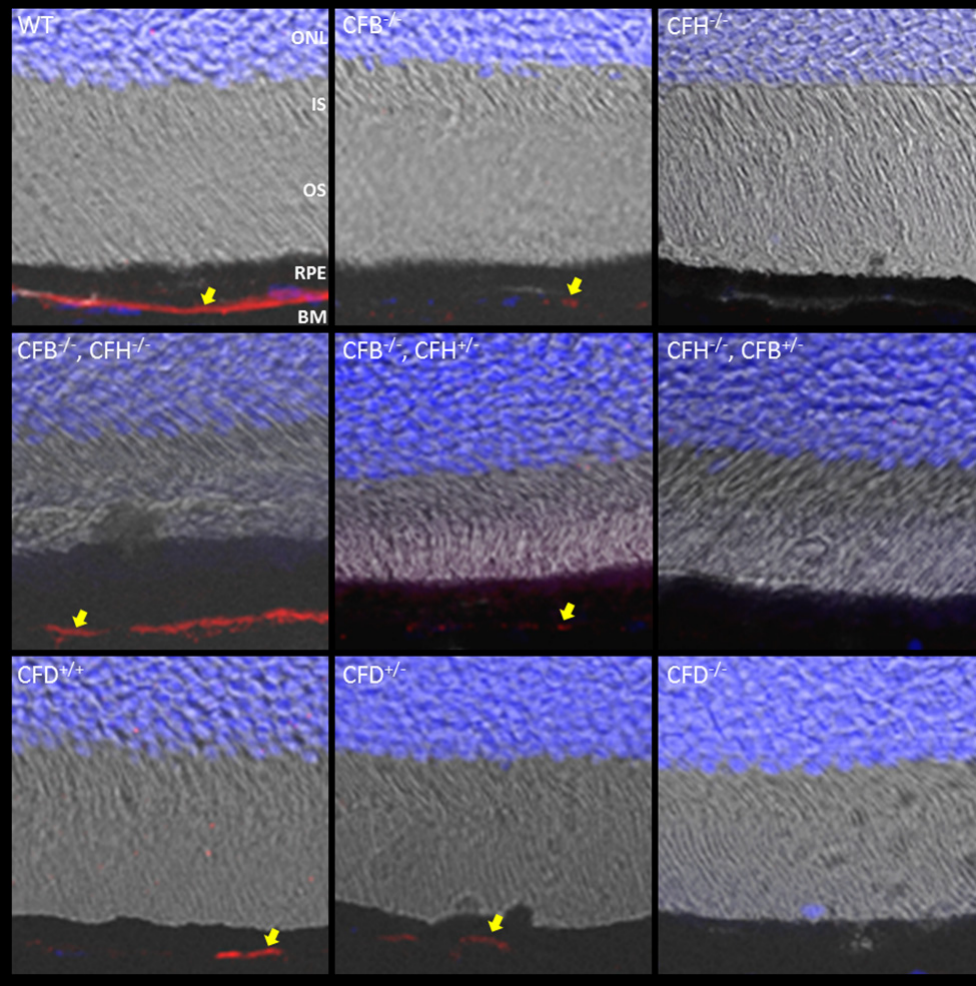
C3 breakdown products in RPE/Bruch’s are absent in *Cfh*^-/-^ mice but restored in the double knock-out *Cfb*^-/-^*/Cfh*^-/-^ mouse. 12 μm PFA fixed sections were taken of mouse retinas of indicated genotypes at 12 months. Sections were stained for C3b/iC3b/C3c (red) and nuclei (blue). Z-stacks were imaged using confocal microscopy and merged to form a maximum intensity projection (MIP). MIPs were merged with digital interference contrast and arrows highlight the presence of C3 breakdown products in RPE/Bruch’s. Scale bars = 50 μm. Images are representative of n = 6.

Activation of the complement system leads to the breakdown of C3 into C3b, which in turn can be further degraded by CFI and CFH into iC3b and C3c. To analyse the presence of these breakdown products in the retina, sections were stained with an antibody specific to C3b/iC3b/C3c (Fig 4). In WT mice, these breakdown products were evident at the basal surface of the RPE, but in contrast to whole C3 none were visible within the retinal blood vessels in WT or any of the mutant strains. Where CFH was deleted in *Cfh*^-/-^ and *Cfh*^-/-^/*Cfb*^+/-^ mice, we observed no staining for C3b/iC3b/C3c consistent with the lack of circulating C3. Where CFB was deleted in *Cfb*^-/-^ and *Cfb*^-/-^/*Cfh*^+/-^ mice we observed only faint punctate staining for C3b/iC3b/C3c probably due to the inability of little C3 convertase to form in the absence of CFB. Surprisingly where both CFH and CFB were deleted in the *Cfb*^-/-^/*Cfh*^-/-^ double knockout mouse, we observed C3b/iC3b/C3c in RPE/Bruch’s in this strain at similar levels to WT. In the *Cfd*^-/-^ mouse, the C3b/iC3b/C3c staining pattern followed that of C3, with no C3b/iC3b/C3c detectable, and weak staining in the *Cfd*^+/-^ mouse. To aid comparison of the different genotypes, qualitative analysis of the staining of C3 and its breakdown products is summarised in Table 1, together with the actual values of circulating C3 in the serum.

**Table 1:** Values of circulating C3 (mg/L ± SEM) are presented for each genotype. N = 6 for all genotypes except *Cfd*^-/-^ where n = 5. Staining intensity for C3 and iC3b in the RPE/Bruch’s membrane was scored as follows: +++ = strong, ++ = moderate, + = weak,— = not detected.

Genotype	C3 in serum (mg/L)	C3 in RPE/Bruch’s	iC3b in RPE/Bruch’s
WT	495 ± 117	+++	+++
*Cfb*^-/-^	749 ± 146	+	+
*Cfh*^-/-^	16.5 ± 2.6	-	-
*Cfb*^-/-^/*Cfh*^-/-^	350 ± 30.3	++	+++
*Cfb*^+/-^/*Cfh*^-/-^	31.9 ± 4.4	-	-
*Cfh*^+/-^/*Cfb*^-/-^	401 ± 111	+	+
*Cfd*^+/+^	415 ± 89	++	++
*Cfd*^+/-^	390 ± 51	++	+
*Cfd*^-/-^	354 ± 91	+++	-

**Fig 5:**
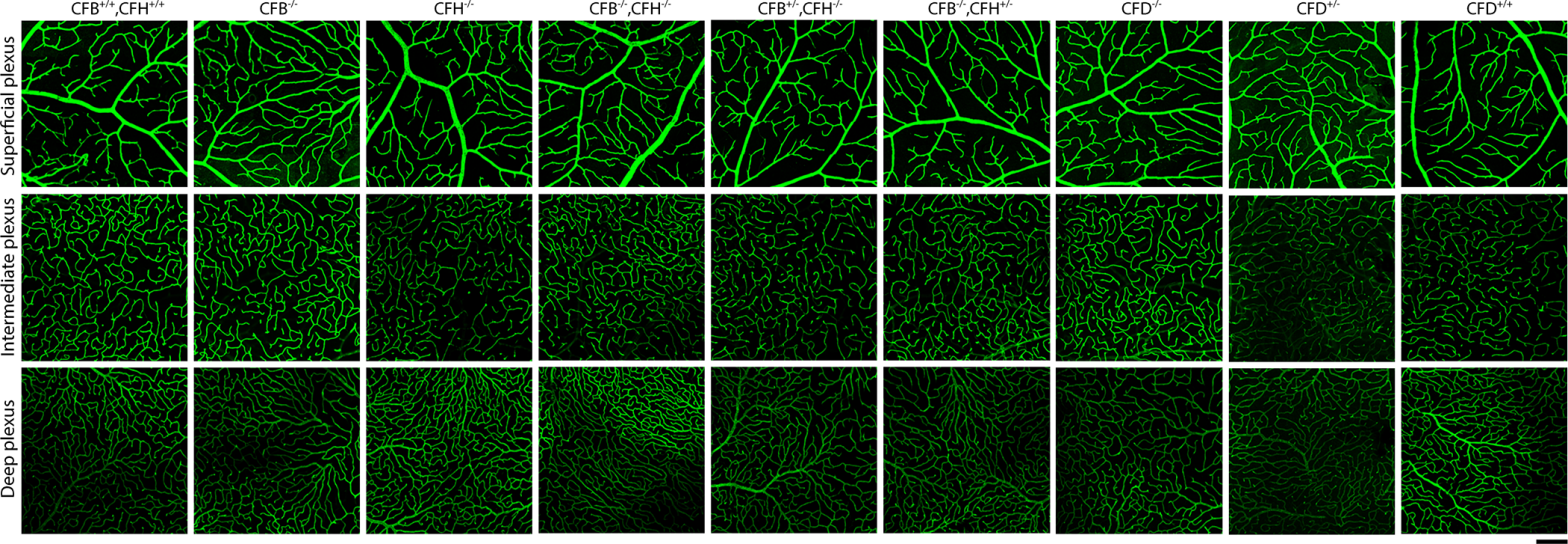
Organisation of the retinal blood vessels is unaffected by the loss of *Cfb* or *Cfh*. Neuroretinas from mice of indicated genotypes aged 12 months were flatmounted and fixed in methanol. Vessels were stained for Collagen IV (green) and z-stacks imaged using confocal microscopy. Images represent a maximum intensity projection of each plexus ranging from 8–12 μm for the inner, intermediate and deep plexuses. Scale bars = 100 μm. Images are representative of n = 3.

It has been previously reported, by fluorescein angiography, that retinal blood vessels in the deep plexus of 1 year *Cfh*^-/-^ mice were withered due to the accumulation of C3 breakdown products on blood vessel walls [21]. Here we failed to detect C3 breakdown products anywhere in the retina (data not shown), but we nevertheless examined retinal vessel morphology and density by immunostaining neuroretinal flatmounts for collagen IV (Fig 5). In all genotypes tested, each retinal vessel plexus was morphologically comparable to that of WT mice with no sign of withering vessels or other vascular abnormalities. The absence of a distinct vascular phenotype in this study is consistent with the majority of reports of complement mutant mice, perhaps indicating that secondary environmental factors such as microbiome or diet may modulate the phenotype in some instances.

## Discussion

CFB and CFH are key mediators in the alternative pathway of complement activation. CFB is required for the formation of the C3 convertase whereas CFH is a key regulator of C3 convertase formation, and also acts as a co-factor for CFI in the breakdown of C3b. Investigating the roles of these proteins within the retina is of particular interest since mutations in both genes have been identified as either protective in the case of CFB (R32Q) [9] or associated with increased risk of developing AMD for CFH (Y402H) [1–4]. It has been shown in the mouse that both proteins are involved in choroidal neovascularisation, in that reduction in CFB has a protective effect upon laser-induced injury whereas loss of CFH leads to enhanced pathological choroidal neovascularisation [11,12,22]. These observations are consistent with the alternative pathway of complement activation playing a role in the development of vascular pathology. Both proteins are present in serum but whether they access the retina from the circulation is unknown. Within the retina, a potentially important local source of CFB and CFH is the RPE, and the expression of both proteins by RPE cells has been reported to increase in response to inflammatory stimuli [10,23].

Several studies have been performed on the retinas of *Cfh*^-/-^ mice [5–7,20,21]. We previously showed functional changes in scotopic electroretinography (ERG) that were mild at 1 year and more pronounced by 2 years. Loss of CFH also led to structural changes in the RPE, photoreceptors and Bruch’s membrane. Overall these studies suggest that the retina requires CFH for complement homeostasis and normal visual function. However, in interpreting these results in the context of CFH function, it must be kept in mind that a confounding feature of these mice is the almost total absence of C3 and CFB from the serum due to its non-regulated breakdown by the absence of CFH in the fluid phase [17]. Indeed, the complete absence of C3 in 1 year old *Cfh*^-/-^/*C3*^-/-^ double knock-out mice causes more pronounced visual dysfunction than in *C3*^-/-^ or *Cfh*^-/-^ single knock-out mice at this age, suggesting that CFH is required for more than just the regulation of C3 convertase formation and breakdown [20]. In patients with AMD, C3 in the serum is not depleted [24,25] and therefore studying the effect of the loss of CFH when C3 is present is of interest. Here we have partially addressed this problem by crossing *Cfb*^-/-^ mice with *Cfh*^-/-^ mice to create the double knockout, and show that in these animals the level of C3 in the retina, as judged by immunostaining, and in the plasma measured by ELISA, is approximately equivalent to that observed in control mice.

Our results show that the loss of CFB, CFH or both proteins together does not lead to any significant anatomical or morphological changes in the retina, at least in the first year of life. This suggests that at 12 months, and in the absence of pathological challenge, infection or environmental stress, the retina is stable and healthy in the absence of a functional alternative pathway. In a recent microarray study of the retinas of young, 7–8 week *Cfh*^-/-^ mice, we observed no significant changes at the transcriptional level in any complement regulatory genes [6]. Examination of the retinal vasculature and microglial infiltration (not shown) similarly revealed no significant differences between genotypes. As expected, the main consequences of deleting CFB, CFH or both proteins, were in relation to the levels of C3 and its breakdown products, C3b, iC3b and C3c, in the retina. Our data confirm those of Pickering et al., who first reported that C3 is no longer present in the serum in *Cfh*^-/-^ mice [17]. The loss of C3 from the basal surface of the RPE in *Cfh*^-/-^ and *Cfh*^-/-^/*Cfb*^+/-^ mice, when C3 is low in the serum, suggests that the C3 that deposits at this location in control, *Cfb*^-/-^ and *Cfb*^-/-^/*Cfh*^+/-^ mice originates from the serum.

Without CFB, C3 convertases are no longer able to form via the alternative pathway and therefore C3 cannot be broken down unless the classical or lectin pathways become activated. Indeed we did see immunostaining of C3 in the retinal vasculature and Bruch’s membrane of the *Cfb*^-/-^ mice. Low levels of C3 were also present along the basal surface of the RPE reinforcing the notion that these C3 deposits are derived from the serum. However, in contrast to WT animals, C3 breakdown products were almost undetectable in the *Cfb*^-/-^ and *Cfb*^-/-^/*Cfh*^+/-^ mice, consistent with C3 convertases being prevented from forming. In the double knock-out mice the loss of both CFH and CFB restored C3 expression to levels similar to those observed in WT or *Cfb*^-/-^ mice. But without the ability to form C3 convertases via the alternative pathway, the regulatory role of CFH becomes redundant. Despite this, in the *Cfb*^-/-^/*Cfh*^-/-^ mice there was significant immunostaining of C3 breakdown products at the basal surface of the RPE suggesting that in these mice, C3b is generated by either the lectin or classical pathway. We can speculate that the loss of CFH in addition to CFB causes an imbalance that is not present in C*fb*^-/-^ mice. When CFB expression was partially restored in the *Cfh*^-/-^/*Cfb*^+/-^ mice C3 expression in the retina was again absent as in the *Cfh*^-/-^ mice. This is because with the restoration of CFB, C3 convertases are again able to form, but without CFH to regulate them, all the C3 would be broken down. As in the *Cfh*^-/-^ mice, without C3 on the basal surface of the RPE, no breakdown products were detected at this site.

We included CFD mutant mice in our analysis as there are few ocular studies that have used this strain, despite CFD being the key serine protease that cleaves C3bB to form the C3 convertase C3bBb, and with the CFD blocking antibody Lampalizumab now in trials for dry AMD [13,14]. Interestingly, we observed particularly intense staining of C3 on the basal RPE/Bruch’s in the *Cfd*^-/-^ mouse retina, even though these mice have normal serum levels of C3, suggesting that production of CFD by the RPE might provide local control of C3 build-up under normal circumstances. It was not surprising that we were unable to detect C3 breakdown products on RPE/Bruch’s since without CFD, the C3 convertase was not able to form via the alternative pathway. In future studies it would be interesting to generate a *Cfd*^-/-^/*Cfh*^-/-^ double knock-out mouse to see whether loss of CFH as in the *Cfb*^-/-^/*Cfh*^-/-^ mouse causes activation of classical or lectin pathways and restores C3 breakdown at the RPE/Bruch’s surface. In summary we have shown, using a range of single and double null mutant mice, that the alternative pathway of complement activation is critical in regulating C3 activation on RPE/Bruch’s. We also show that regardless of genotype the effects of disrupting *Cfb*, *Cfh* and *Cfd* on retinal anatomy are mild at one year of age. Some of our observations cannot be readily explained on the basis of systemic complement activation alone, and hint instead at RPE-derived pools having specific roles that may be important in the development of AMD.
